# Factors affecting athletes’ motor behavior after the observation of scenes of cooperation and competition in competitive sport: the effect of sport attitude

**DOI:** 10.3389/fpsyg.2015.01648

**Published:** 2015-10-28

**Authors:** Elisa De Stefani, Doriana De Marco, Maurizio Gentilucci

**Affiliations:** Department of Neuroscience, University of ParmaParma, Italy

**Keywords:** scenes of cooperation and competition, expert athletes, cooperative/competitive attitude, kinematics, social interaction

## Abstract

**Aim:** This study delineated how observing sports scenes of cooperation or competition modulated an action of interaction, in expert athletes, depending on their specific sport attitude.

**Method:** In a kinematic study, athletes were divided into two groups depending on their attitude toward teammates (cooperative or competitive). Participants observed sport scenes of cooperation and competition (basketball, soccer, water polo, volleyball, and rugby) and then they reached for, picked up, and placed an object on the hand of a conspecific (giving action). Mixed-design ANOVAs were carried out on the mean values of grasping-reaching parameters.

**Results:** Data showed that the type of scene observed as well as the athletes’ attitude affected reach-to-grasp actions to give. In particular, the cooperative athletes were speeded when they observed scenes of cooperation compared to when they observed scenes of competition.

**Discussion:** Participants were speeded when executing a giving action after observing actions of cooperation. This occurred only when they had a cooperative attitude. A match between attitude and intended action seems to be a necessary prerequisite for observing an effect of the observed type of scene on the performed action. It is possible that the observation of scenes of competition activated motor strategies which interfered with the strategies adopted by the cooperative participants to execute a cooperative (giving) sequence.

## Introduction

A growing number of behavioral and neurophysiological studies have demonstrated that perception and action have a common coding (Rizzolatti and Craighero, 2004; Rizzolatti et al., 2014). The concept of affordances, as originally postulated by Gibson (1978), refers to the possibilities for action that emerge from the interactions of an organism with its environment. Further evidence has demonstrated that activation of affordances is modulated not just by the physical properties of objects, but also by the social context in which an action is performed (Mason and Mackenzie, 2005; Georgiou et al., 2007; Meulenbroek et al., 2007; Becchio et al., 2008; Sartori et al., 2009; Ferri et al., 2010, 2011, 2014; Innocenti et al., 2012). Indeed, social behavior during interaction with conspecifics (i.e., different intentions of the agent or the observer) can interact with affordance instantiation and modify the kinematics of the actions. The ability to read others’ intentions plays an important role in sports, as athletes need to perceive the action capabilities of their opponents and their teammates in order to be aware of ever-changing opportunities for action afforded by a sport situation (Passos et al., 2009; Correia et al., 2012; Vilar et al., 2012).

Throughout the course of a game, players can implement both defensive and offensive behaviors. It is possible that these behaviors lead to the development of certain skills to either cooperate or compete with teammates. Moreover, with the development of expertise in a sport, athletes improve specific patterns of interaction, that is, a personal predisposition to be more cooperative or competitive toward their teammates. We refer to these specific strategies using the term “attitude”: a predisposition toward a specific motor behavior in response to an actual sport setting.

It is well known that the observation of an action activates a process of simulation (Buccino et al., 2004b). For transitive actions (directed upon an object), the same act done by another agent corresponds to the activation of an internal motor representation of that act. This simulation is used to understand the goal of the movement (Buccino et al., 2001, 2004a; Iacoboni et al., 2005). In the case of intransitive actions, the simulation is mainly used to understand the intention of the agent (Fadiga et al., 1995; Buccino et al., 2001; Rizzolatti and Craighero, 2004). In summary, the simulation of an observed action allows one to recognize the goal of the observed movement, to infer others’ intentions, and to predict the agent’s next act. Moreover, this mechanism of intention understanding can modulate a further self-generated action. In other words, the observation of an action can influence the motor response of a subsequent action. This happens often in a sport context: actions are frequently executed in the presence of another acting individual whose intentions can be cooperative or competitive. Consequently, the observation of sport scenes of cooperation and competition can differently affect the subsequent action of the observer. We hypothesized that this effect would enhance the cooperative and competitive attitude of an athlete. Athletes that are attuned to simulating sportive actions can be greatly affected, compared to non-athletes, in the execution of a subsequent action after observing sportive scenes of cooperation and competition.

We extended our research to sport expertise by considering athletes’ attitudes (cooperative versus competitive). Two main issues were examined in this study: firstly, we were interested in ascertaining whether the sole observation of well-known sport actions in a context of cooperation or competition could influence the kinematics of a cooperative social interaction with a conspecific (giving action). Specifically, we expected that the observation of an action of cooperation could facilitate a successive executed action of cooperation, making the participant’s movement faster. On the other hand, the observation of an action of competition could interfere with the participant’s action of cooperation, probably slowing down the movement. Secondly, we were interested in investigating how the kinematics of athletes’ actions can be modulated not only by the observation of a specific cooperative/competitive sport action, but also by the attitude of the participants. We hypothesized that the interaction between the participant’s attitude (cooperative or competitive) and the type of sport actions observed (an action of cooperation or an action of competition) could modulate a successive motor response, affecting the kinematics of reach–grasp movements performed by participants. Specifically, we expected that the congruence between the participant’s attitude (e.g., cooperative attitude) and an observed action (e.g., action of cooperation) could facilitate the execution of a successive movement toward a conspecific, making the participant’s action faster. On the other hand, we expected that the incongruence matching (e.g., cooperative attitude versus the observation of an action of competition) could interfere with a successive interaction with a conspecific, presumably slowing down the movement. In other words, we expected facilitation only when the attitude of the participant was congruent with the type of observed action.

## Materials and Methods

### Participants

Twenty right-handed undergraduate students (9 male, 11 female) between the ages of 20 and 28 years (mean = 21.6, *SD* = 2.5) took part in the present experiment. They all practiced a sport more than three times per week (*SD* = 1.7) and they all had experience in one or more of the team sports selected in this study (**Table 1**). Handedness was assessed through the Edinburgh Inventory (Oldfield, 1971). The participants were students of the degree course of Motor Sciences, Sport and Health (University of Parma) and practiced team sports at the competitive level. Before being included in our study, the participants completed a questionnaire to collect information about what sport they practiced; which position they played; and whether they felt more cooperative with their peers than competitive toward their opponents during a game (see Data Sheet 1). The participants were divided into two groups (cooperative and competitive group) according to their answers. In the competitive group, we included only participants that had clearly exhibited competitive behavior during matches (13 competitive athletes). We used the same criteria for athletes included in the cooperative group (seven cooperative athletes). We excluded the uncertain participants. All participants provided a written informed consent to participate in the study, which has been approved by the local ethical committee (Comitato Etico per Parma) and has been conducted according to the principles expressed in the Declaration of Helsinki.

**Table 1:** Participants’ characteristics.

Participants	Age	Attitude	Sport	Frequency	Sex	Expertise
1	20	cooperative	basket	>3 days, a week	M	more than 1 years
2	20	competitive	volleyball	>4 days a week	F	more than 1 years
3	20	competitive	volleyball	>4 days, a week	F	more than 1 years
4	28	competitive	water polo	>4 days a week	F	more than 1 years
5	20	competitive	water polo	>4 days a week	F	more than 1 years
6	20	cooperative	soccer	>3 days a week	M	more than 1 years
7	21	competitive	volleyball	>3 days a week	F	more than 1 years
8	20	competitive	volleyball	>4 days a week	F	more than 1 years
9	20	cooperative	volleyball	>4 days, a week	F	more than 1 years
10	21	competitive	soccer	>4 days a week	M	more than 1 years
11	20	competitive	soccer	>4 days, a week	M	more than 1 years
12	21	cooperative	rugby	>4 days a week	M	more than 1 years
13	21	cooperative	volleyball	>3 days a week	F	more than 1 years
14	20	competitive	volleyball	>4 days a week	F	more than 1 years
15	20	competitive	basket	>3 days a week	F	more than 1 years
16	21	cooperative	soccer	>4 days a week	M	more than 1 years
17	21	competitive	soccer	>4 days, a week	M	more than 1 years
18	26	cooperative	soccer	>4 days a week	M	more than 1 years
19	26	competitive	basket	>4 days, a week	F	more than 1 years
20	25	competitive	soccer	>4 days a week	M	more than 1 years


### Apparatus, Stimuli, and Procedure

**FIGURE 1:**
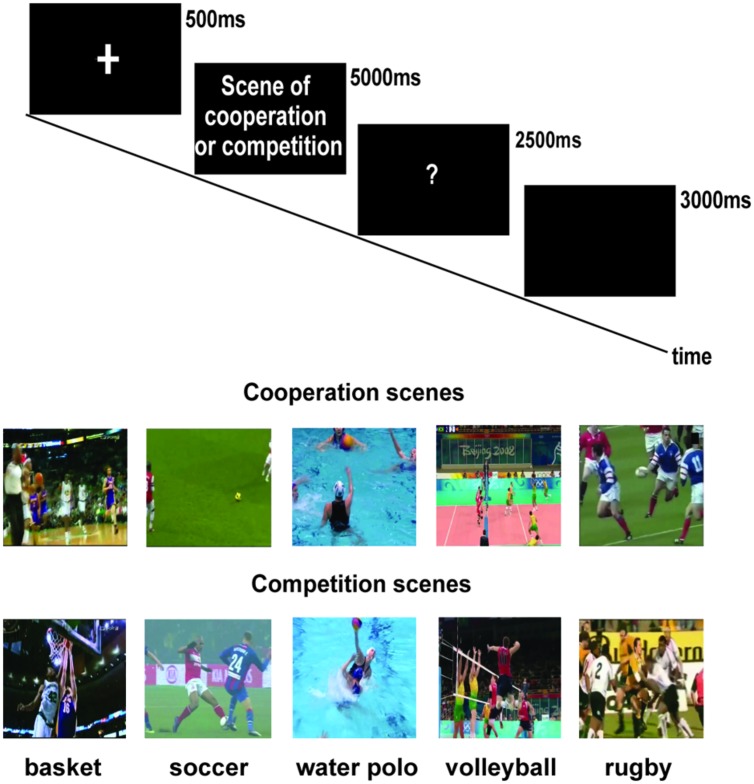
**Procedure and stimuli presented in the experiment**.

The participants sat comfortably in front of a table on which they placed their right hand with the thumb and index finger in pinch position starting position (SP). SP was located along the participants’ mid-sagittal plane and was 27 cm away from their chest. An experimenter was seated next to the participant, and she held the palm of her right hand in the supine position (request position). A computer display was placed on a table plane at a distance of 60 cm from the body of the participant sitting in front of it. A wooden cube (∼2 cm × 2 cm × 2 cm) was placed at the center of the table 20 cm in front of participant’s SP. Stimuli were presented on the computer display using software developed via MATLAB version 7.7 (R2008b). The stimuli were short videos downloaded from the Internet replicating real matches. Each video lasted five seconds. We selected videos based on the following criterion: (a) the action would involve coordinated sports action among athletes of the same team, or (b) two or more athletes from two different teams would come into contact with each other. Consequently, the actions defined “actions of cooperation”-reproduced situations in which athletes of the same team cooperated in an action of the game (e.g., in volleyball, a pass ball between setter and hitter, see **Figure 1**). In the “actions of competition”-reproduced situations, two athletes from two different teams were opposed (e.g., in a soccer match, the attacker tries to score a goal and the defender marks him). Selected scenes reproduced sports actions in which the participants were experts—that is, five cooperation and five competition scenes from the following sports: basketball, soccer, water polo, volleyball, and rugby (**Figure 1**). In total, 50 scenes were presented. After the presentation of a fixation cross (500 ms), participants viewed one of the 10 videos that lasted 5,000 ms. As soon as they understood whether the action was one of cooperation or competition, they were required to reach for, pick up, and place the wooden cube on the experimenter’s hand (giving action). The participants grasped the cube with their fingers (right hand, precision grip). When a question mark (2,500 ms) appeared on the computer display, the participants were instructed to state out loud whether the just seen action was an action of cooperation or competition (10% catch trials). Subsequently, a black screen was presented (3,000 ms). The participants had to place their hands in SP and then wait for the next trial. In total, the participants responded correctly to the cooperation condition in 99% of the cases and in the competition condition in 99.7% of the cases.

### Data Recording

The movements of the participants’ right arms were recorded using the 3D-optoelectronic SMART system (BTS Bioengineering, Milan, Italy). This system consists of six video cameras that detect infrared reflecting markers (spheres that are 5 mm in diameter) at a sampling rate of 120 Hz. The spatial resolution of the system is 0.3 mm. The infrared reflective markers were attached to the nail of the participants’ right thumbs and index fingers, and another marker was attached to the participants’ right wrists. The markers attached to the thumb and index finger were used to analyze the grasp kinematics, whereas the marker attached to the wrist was used to analyze the kinematics of reaching and lifting. Manual prehension consists of two components: the proximal component (also known as “the reach”), which is the action of carrying the hand toward an object, and “the grasp” component, during which the fingers are opened and shaped before the contact of the hand with the target (Jeannerod, 1984; Jakobson and Goodale, 1991; Gentilucci et al., 2001). The reach transports the hand toward the object (the reaching action makes the hand move toward an object), and its kinematics depend on the target’s extrinsic properties (i.e., location and orientation). The grasp component provides information on how to open, preshape, and close the hand during the reach in relation to the target’s intrinsic properties (i.e., size and shape). The data of the recorded movements was analyzed using software developed via MATLAB version 7.7 (R2008b). Recorded data were filtered using a Gaussian low-pass smoothing filter (∑ = 0.93). The time course of the reach, grasp, and lift was visually inspected: the beginning of the grasp was considered to be the first frame in which the distance between the two markers placed on the right finger tips increased more than 0.3 mm (spatial resolution of the recording system) with respect to the previous frame. The end of the grasp was the first frame after the beginning of the finger closing, in which the distance between the two right fingers decreased less than 0.3 mm with respect to the previous frame. The beginning of the reach was considered to be the first frame during which the displacement of the reach marker along any Cartesian body axis increased more than 0.3 mm with respect to the previous frame. To determine the end of the reach, we calculated the first frame following movement onset separately for the X, Y, and Z axes, in which the X, Y, and Z displacements of the reach marker decreased less than 0.3 mm compared to the previous frame. Then, the frame endpoint temporally closer to the grasp end frame was chosen as the end of the reach. The frame immediately succeeding the reach end was considered as the lift beginning, while the lift end corresponded to the frame in which the highest point of the hand trajectory was reached during lifting. The grasp was studied by analyzing the time course of the distance between the index finger and thumb markers. From a pinch position, the grasp component was constituted of an initial phase of finger opening up to a maximum (maximal finger aperture) followed by a phase of finger closing on the object (Jeannerod, 1988).

We measured the following parameters: reach time, time to peak velocity of reach, peak elevation (trajectory maximal height), grasp time, time to maximal finger aperture, peak velocity of finger opening, time to peak velocity of finger opening, and maximal finger aperture.

### Data Analysis

Participants were divided into two groups (cooperative attitude versus competitive attitude) according to the questionnaire responses. They resulted in 7 cooperative participants and 13 competitive participants (**Table 1**). Because of the difference in sample size between groups, the homogeneity of variance was primarily verified with Levene’s test. Mixed-design ANOVAs were carried out on the mean values of the reaching–grasping parameters (**Table 2**). The within-subject factor was the type of scene (cooperation versus competition) and the between-subject factor was the participants’ attitudes (cooperative versus competitive). In all of the analyses, *post hoc* comparisons were performed using the Newman–Keuls procedure. The significance level was fixed at *p* = 0.05. When a factor was significant, we also calculated the effect size ($\eta_{p}^{2}$). We also carried another mixed-design ANOVA, using gender (male versus female) and type of practiced sport (basketball versus soccer versus water polo versus volleyball versus rugby) as the between-subject factors. All of these final analyses were not significant, and the corresponding *p*-values are reported as **Supplementary Table S1**.

**Table 2:** Mean values and SE of kinematic parameters of reach and grasp action.

	Scene of cooperation				Scene of competition			
		
Kinematic parameters	Cooperative attitude		Competitive attitude		Cooperative attitude		Competitive attitude	
				
	Mean	*SE*	Mean	*SE*	Mean	*SE*	Mean	*SE*
Reach time (ms)	637	47	513	35	686	50	523	37
Time to peak velocity of reach (ms)	287	21	256	15	313	22	258	16
Peak elevation (mm)	94	5	91	4	96	5	93	4
Grasp time (ms)	607	44	512	32	645	44	504	32
Time to maximal finger aperture (ms)	420	36	316	27	455	37	311	27
Peak velocity of finger opening (mm/s)	233	43	305	31	217	14	309	30
Time to peak velocity of finger opening (ms)	208	28	160	21	238	31	152	23
Maximal finger aperture (mm)	78	3	84	2	77	3	83	2


## Results

### Reach

The main factor of the participants’ attitudes was significant. There was a significant difference in reach time between cooperative participants and competitive participants [*F*(1,18) = 5.74, *p* < 0.028; cooperative = 662 ms versus competitive = 518 ms].

**FIGURE 2:**
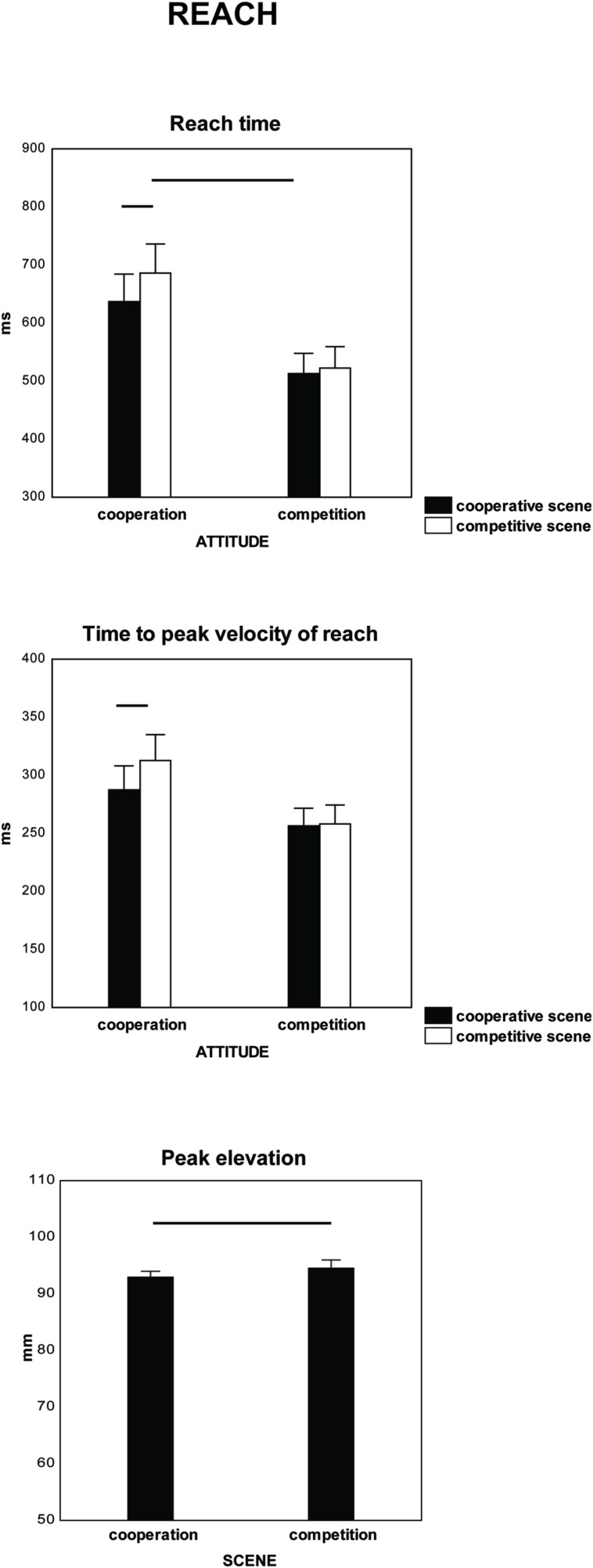
**Parameters of reach (reach time, time to peak velocity of reach, peak elevation (trajectory maximal height) which were significant on Mixed-design ANOVAs.** The within-subjects factor was type of scene (cooperation vs. competition) and the between-subjects factor was participants’ attitude (cooperative vs. competitive). Vertical bars are standard errors (SE).

Factor scene affected reach time and time to peak velocity of reach. Scenes of cooperation induced a decrease in both parameters in comparison with scenes of competition [reach time: *F*(1,18) = 15, $\eta_{p}^{2}$ = 0.45, *p* < 0.00, 575 ms versus 604 ms; time to peak velocity of reach: *F*(1,18) = 6.5, $\eta_{p}^{2}$ = 0.27, *p* < 0.02, 271 ms versus 285 ms]. It is possible that the scenes of cooperation facilitated, and/or the scenes of competition interfered with, the reach (and grasp, see below) because the participants executed a giving (cooperative) action. The interaction between the type of scene and the participants’ attitudes also affected reach time [*F*(1,18) = 6.8, $\eta_{p}^{2}$ = 0.274, *p* < 0.018] and time to peak velocity of reach [*F*(1,18) = 5.01, $\eta_{p}^{2}$ = 0.218, *p* < 0.038, **Figure 2** and **Table 2**]. *Post hoc* comparison showed a significance between types of scene only when the participants were cooperative (reach time: *p* = 0.00037; time to peak velocity of reach: *p* = 0.003). No difference was found between scenes of cooperation and competition when participants were competitive (reach time: *p* = 0.384; time to peak velocity of reach: *p* = 0.827). Finally, scenes of cooperation and competition affected peak elevation differentially [*F*(1,18) = 4.7, $\eta_{p}^{2}$ = 0.208, *p* < 0.043, 93 mm versus 95 mm].

### Grasp

Competitive participants showed a significant decrease in grasp time and time to maximal finger aperture compared to cooperative participants (grasp time: *F*(1,18) = 4.8, *p* < 0.042, 508 ms versus 626 ms; time to maximal finger aperture: *F*(1,18) = 7.5, *p* < 0.013, 314 ms versus 437 ms).

**FIGURE 3:**
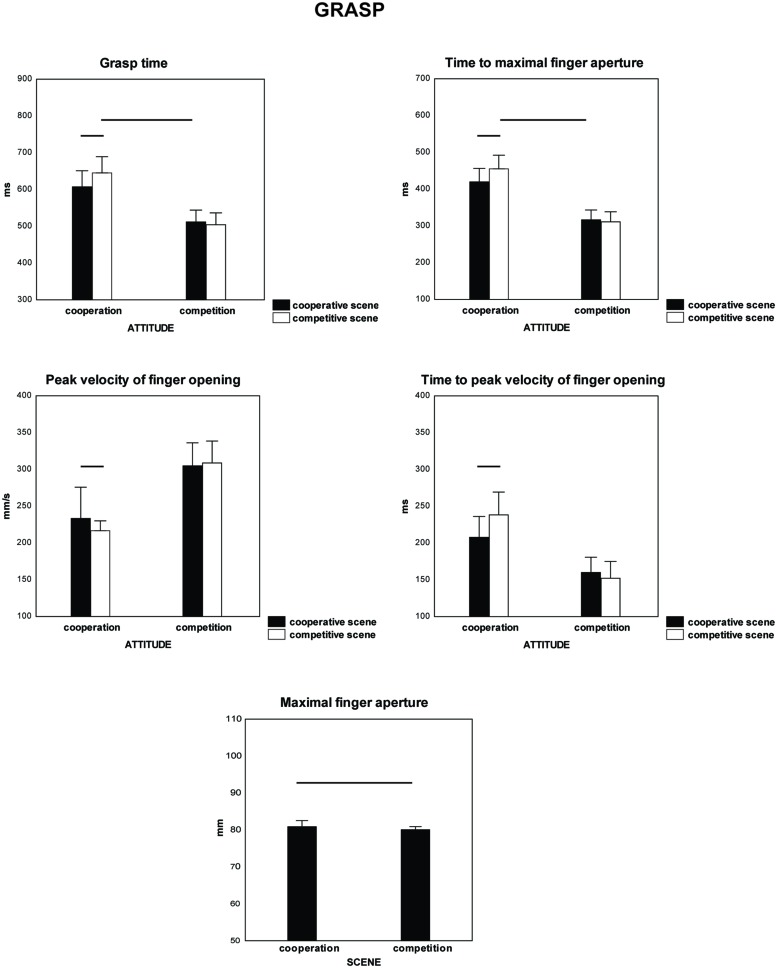
**Parameters of grasp (grasp time, time to maximal finger aperture, peak velocity of finger opening, time to peak velocity of finger opening, maximal finger aperture which were significant on Mixed-design ANOVAs.** The within-subjects factor was type of scene (cooperation vs. competition) and the between-subjects factor was participants’ attitude (cooperative vs. competitive). Vertical bars are SE.

A significant interaction between the factor type of the scene and the participants’ attitudes was found for grasp time [*F*(1,18) = 7.24, $\eta_{p}^{2}$ = 0.287, *p* < 0.015] and time to maximal finger aperture [*F*(1,18) = 6.35, $\eta_{p}^{2}$ = 0.261, *p* < 0.021, **Table 2** and **Figure 3**]. *Post hoc* comparison showed a significant decrease in the parameters for scenes of cooperation only when the participants were cooperative (grasp time: *p* = 0.005; time to maximal finger aperture: *p* = 0.006). No difference was found between the scenes of cooperation and competition presented to competitive participants (grasp time: *p* = 0.533; time to maximal finger aperture: *p* = 0.639). The interaction between the type of scene and the participants’ attitudes showed a trend toward significance for peak velocity of finger opening [*F*(1,18) = 3.88, $\eta_{p}^{2}$ = 0.177, *p* < 0.064] and significance for time to peak velocity of finger opening [*F*(1,18) = 8.69, $\eta_{p}^{2}$ = 0.325, *p* < 0.009]. *Post hoc* comparisons showed a significant decrease in the two parameters in the presence of scenes of cooperation only when they were presented to cooperative participants (peak velocity of finger opening: *p* = 0.037; time to peak velocity of finger opening: *p* = 0.0039). Scenes of cooperation and competition differentially affected maximal finger aperture. Participants opened their fingers to a larger degree when grasping the target after seeing scenes of cooperation compared to competition [*F*(1,18) = 5.2, $\eta_{p}^{2}$ = 0.225, *p* < 0.035; 81 mm versus 80 mm].

In sum, the participants were facilitated (i.e., faster) when executing actions of cooperation after observing actions of cooperation. This occurred only when they had cooperative attitudes. In general, the competitive participants were faster than the cooperative ones.

## Discussion

The aim of the present study was to determine whether and how the matching between the athletes’ attitudes (cooperative and competitive attitude) and the observation of sport scenes (actions of cooperation and competition) could influence the kinematics of a successive social interaction. The participants were all expert athletes in at least one of the team sports selected for this study (basketball, soccer, water polo, volleyball, and rugby; **Figure 1**). Before starting the experiment, the athletes were divided into two groups according to their attitude during a game (cooperative versus competitive attitude; see Materials and Methods). The participants had to observe a sport scene of cooperation or competition before performing a motor sequence. They executed a reach–grasp of an object and placed it in the hand of an experimenter who was sitting close to them (a cooperative giving action). Our expectation was that both the participants’ attitudes and the type of scene would influence the sequence kinematics.

Firstly, we observed an effect of attitude. The competitive participants were faster than the cooperative ones during the action execution regardless of the observed scene. A possible explanation for this finding is that competitive athletes are generally faster in performing an action than cooperative athletes are. Alternatively, the cooperative athletes could be less competitive, and for this reason, they are slower in performing an action with respect to competitive athletes. A further possible explanation is that the lack of any effect when the scenes of cooperation and competition were presented to the competitive athletes might depend on the inability of these athletes to adopt strategies that are suitable to successfully execute the giving sequence toward a conspecific.

Secondly, we observed an interaction effect between the athletes’ attitudes and the type of scene on the reach–grasp temporal parameters. The cooperative participants were faster in their movement when they observed scenes of cooperation, subsequently executing the giving action. On the contrary, these athletes were slower when they observed scenes of competition.

It is possible that the observed action could have been automatically mapped onto participants’ motor system, resulting in a facilitation of functionally similar actions. In other words, the observed scene probably acted as a prime stimulus for the subsequent executed action. This facilitation effect would have been present when the participants observed a scene of cooperation and then had to perform a cooperative motor sequence toward a conspecific. On the other hand, there would have been an interference effect when the participants observed a scene of competition and had to perform a cooperative motor sequence (Chartrand and Bargh, 1999; Brass et al., 2000, 2001; Flanagan and Johansson, 2003; Kilner et al., 2003; Sebanz et al., 2003, 2006; Newman-Norlund et al., 2007; Liepelt et al., 2008; Bekkering et al., 2009). However, the competitive participants did not show any effect. The fact that only the cooperative participants were affected by the type of scene they observed suggests that the effect was more complex than a simple priming.

Only when there was congruence between the attitude and the observed action was it possible to observe changes in the kinematics of a giving action. Specifically, in the case of congruence (i.e., cooperative attitude and observation of a scene of cooperation), the kinematics of the cooperative participants sped up, whereas in the case of incongruence, they slowed down. On the contrary, the competitive athletes seemed to not be directly affected by the experimental conditions. A possible explanation of this result is that they were already faster and, for this reason, the difference between actions of cooperation and competition did not emerge. What would happen if the competitive athletes had to perform a competitive action (e.g., grasp the target and move it away from the conspecific)? Might we expect that the competitive athletes would be faster if they have just observed a scene of competition and slowed down in the case of cooperation? We cannot exclude this possibility. However, we suppose that an action of competition would be performed quickly in order to take away the object as quickly as possible (Georgiou et al., 2007). Consequently, it is possible that the speed of this action may prevent us from observing any effect. However, we believe that deepening these aspects could have interesting implications. For this reason, in future experiments, it would be useful to include a control action, for example, asking the participant to move an object away from the conspecific in order to measure how observing scenes of cooperation and competition affects a competitive action.

Deepening and extending the present results with future studies could have interesting implications for training athletes through the observation of specific sport scenes. For an example, it is possible to speculate that competitive athletes, who were found to be faster in their responses, could be trained to be even faster in their movements through the vision of competitive sport actions.

Finally, we are aware of some limitations in this study. First, we chose to measure the participants’ attitudes using a dichotomous item instead of a continuous variable. The reason for our choice was that we wanted to compare the effects of the cooperative and competitive attitude to the videos that were dichotomous (scenes of cooperation and competition). To solve this problem, we included only the athletes who clearly expressed a well-defined position with respect to their attitude, excluding those who were uncertain. Future studies might include sport scenes classified with various degrees of cooperativeness and competitiveness. In this way, it would be possible to compare the participants’ attitudes to the observed scenes in a continuous dimension. Another severe limitation in this study is the very small sample used and the different numbers of males and females and of cooperative and competitive participants (see **Table 1**). For this reason, these findings cannot be generalized to the broader community based on this study alone. In future studies, a larger sample should be used to successfully replicate the present results.

Another important limitation of this study is that we did not use a control group. Future studies might include, for example, a non-athlete group. However, athletes have become more attuned to cooperative and competitive sport situations than non-athletes throughout the course of their sports training. A non-athlete participant group does not have this expertise, so it could be difficult to control the reason why they defined themselves as cooperative or competitive. Another possibility could be to use athletes that play an individual sport, such as dancing or skiing, as a control group. Nevertheless, attention should be paid to their inclusion in the group of cooperative or competitive participants. Finally, another limitation of this study is the lack of a baseline condition against which we could have compared the participants’ kinematics after watching the cooperative and competitive scenes. This aspect is very important, as by including a baseline condition, we could have verified whether watching the different scenes facilitated or interfered with the cooperative participants. Future studies should include a neutral observed scene, for example, a sportive action with just one athlete (e.g., just one soccer player dribbling the ball) as a baseline.

## Conflict of Interest Statement

The authors declare that the research was conducted in the absence of any commercial or financial relationships that could be construed as a potential conflict of interest.

## References

[B1] Becchio C. Sartori L. Bulgheroni M. Castiello U. (2008). Both your intention and mine are reflected in the kinematics of my reach-to-grasp movement. *Cognition* 106 894–912. 10.1016/j.cognition.2007.05.004 17585893

[B2] Bekkering H. de Bruijn E. R. A. Cuijpers R. H. Newman-Norlund R. Van Schie H. T. Meulenbroek R. (2009). Joint action: neurocognitive mechanisms supporting human interaction. *Top. Cogn. Sci.* 1 340–352. 10.1111/j.1756-8765.2009.01023.x 25164937

[B3] Brass M. Bekkering H. Prinz W. (2001). Movement observation affects movement execution in a simple response task. *Acta Psychol. (Amst.)* 106 3–22. 10.1016/S0001-6918(00)00024-X 11256338

[B4] Brass M. Bekkering H. Wohlschläger A. Prinz W. (2000). Compatibility between observed and executed finger movements: comparing symbolic, spatial, and imitative cues. *Brain Cogn.* 44 124–143. 10.1006/brcg.2000.1225 11041986

[B5] Buccino G. Binkofski F. Fink G. R. Fadiga L. Fogassi L. Gallese V. (2001). Action observation activates premotor and parietal areas in a somatotopic manner: an fMRI study. *Eur. J. Neurosci.* 13 400–404. 10.1046/j.1460-9568.2001.01385.x 11168545

[B6] Buccino G. Binkofski F. Riggio L. (2004a). The mirror neuron system and action recognition. *Brain Lang.* 89 370–376. 10.1016/S0093-934X(03)00356-0 15068920

[B7] Buccino G. Lui F. Canessa N. Patteri I. Lagravinese G. Benuzzi F. (2004b). Neural circuits involved in the recognition of actions performed by nonconspecifics: an fMRI study. *J Cogn. Neurosci.* 16 114–126. 10.1162/089892904322755601 15006041

[B8] Chartrand T. L. Bargh J. A. (1999). The chameleon effect: the perception-behavior link and social interaction. *J. Pers. Soc. Psychol.* 76 893–910. 10.1037/0022-3514.76.6.893 10402679

[B9] Correia V. Araújo D. Duarte R. Travassos B. Passos P. Davids K. (2012). Changes in practice task constraints shape decision-making behaviours of team games players. *J. Sci. Med. Sport* 15 244–249. 10.1016/j.jsams.2011.10.004 22153899

[B10] Fadiga L. Fogassi L. Pavesi G. Rizzolatti G. (1995). Motor facilitation during action observation: a magnetic stimulation study. *J. Neurophysiol.* 73 2608–2611. 7666169 10.1152/jn.1995.73.6.2608

[B11] Ferri F. Busiello M. Campione G. C. De Stefani E. Innocenti A. Romani G. L. (2014). The eye contact effect in request and emblematic hand gestures. *Eur. J. Neurosci.* 39 841–851. 10.1111/ejn.12428 24289090

[B12] Ferri F. Campione G. C. Dalla Volta R. Gianelli C. Gentilucci M. (2010). To me or to you? When the self is advantaged. *Exp. Brain Res.* 203 637–646. 10.1007/s00221-010-2271-x 20445966

[B13] Ferri F. Campione G. C. Dalla Volta R. Gianelli C. Gentilucci M. (2011). Social requests and social affordances: how they affect the kinematics of motor sequences during interactions between conspecifics. *PLoS ONE* 6:e15855 10.1371/journal.pone.0015855 PMC3026044 21283642

[B14] Flanagan J. R. Johansson R. S. (2003). Action plans used in action observation. *Nature* 424 769–771. 10.1038/nature01861 12917683

[B15] Gentilucci M. Benuzzi F. Gangitano M. Grimaldi S. (2001). Grasp with hand and mouth: a kinematic study on healthy subjects. *J. Neurophysiol.* 86 1685–1699. 11600632 10.1152/jn.2001.86.4.1685

[B16] Georgiou I. Becchio C. Glover S. Castiello U. (2007). Different action patterns for cooperative and competitive behaviour. *Cognition.* 102 415–433. 10.1016/j.cognition.2006.01.008 16516188

[B17] Gibson J. J. (1978). The ecological approach to the visual perception of pictures. *Leonardo* 11 227–235.

[B18] Iacoboni M. Molnar-Szakacs I. Gallese V. Buccino G. Mazziotta J. C. Rizzolatti G. (2005). Grasping the intentions of others with one’s own mirror neuron system. *PLoS Biol.* 3:e79 10.1371/journal.pbio.0030079 PMC1044835 15736981

[B19] Innocenti A. De Stefani E. Bernardi N. F. Campione G. C. Gentilucci M. (2012). Gaze Direction and Request Gesture in Social Interactions. *PLoS ONE* 7:e36390 10.1371/journal.pone.0036390 PMC3365068 22693550

[B20] Jakobson J. S. Goodale M. A. (1991). Factors affecting higher-order movement planning: a kinematic analysis of human prehension. *Exp. Brain Res.* 86 199–208. 10.1007/BF00231054 1756790

[B21] Jeannerod M. (1984). The timing of natural prehension movements. *J. Mot. Behav.* 16 235–254. 10.1080/00222895.1984.10735333 15151851

[B22] Jeannerod M. (1988). *The Neural and Behavioural Organization of Goal-Directed Movements.* New York, NY, US: Clarendon Press.

[B23] Kilner J. M. Paulignan Y. Blakemore S. J. (2003). An interference effect of observed biological movement on action. *Curr. Biol.* 13 522–525. 10.1016/S0960-9822(03)00165-9 12646137

[B24] Liepelt R. Von Cramon D. Y. Brass M. (2008). How do we infer others’ goals from non-stereotypic actions? The outcome of context-sensitive inferential processing in right inferior parietal and posterior temporal cortex. *Neuroimage.* 43 784–792. 10.1016/j.neuroimage.2008.08.007 18773963

[B25] Mason A. H. Mackenzie C. L. (2005). Grip forces when passing an object to a partner. *Exp. Brain Res.* 163 173–187. 10.1007/s00221-004-2157-x 15761722

[B26] Meulenbroek R. G. J. Bosga J. Hulstijn M. Miedl S. (2007). Joint-action coordination in transferring objects. *Exp. Brain Res.* 180 333–343. 10.1007/s00221-007-0861-z 17256158 PMC1914230

[B27] Newman-Norlund R. D. Noordzij M. L. Meulenbroek R. G. J. Bekkering H. (2007). Exploring the brain basis of joint action: co-ordination of actions, goals and intentions. *Soc. Neurosci.* 2 48–65. 10.1080/17470910701224623 18633806

[B28] Oldfield R. C. (1971). The assessment and analysis of handedness: the Edinburgh inventory. *Neuropsychologia* 9 97–113. 10.1016/0028-3932(71)90067-4 5146491

[B29] Passos P. Araújo D. Davids K. Gouveia L. Serpa S. Milho J. (2009). Interpersonal pattern dynamics and adaptive behavior in multiagent neurobiological systems: conceptual model and data. *J. Mot. Behav.* 41 445–459. 10.3200/35-08-061 19482724

[B30] Rizzolatti G. Cattaneo L. Fabbri-Destro M. Rozzi S. (2014). Cortical mechanisms underlying the organization of goal-directed actions and mirror neuron-based action understanding. *Physiol. Rev.* 94 655–706. 10.1152/physrev.00009.2013 24692357

[B31] Rizzolatti G. Craighero L. (2004). The mirror-neuron system. *Annu. Rev. Neurosci.* 27 169–192. 10.1146/annurev.neuro.27.070203.144230 15217330

[B32] Sartori L. Becchio C. Bulgheroni M. Castiello U. (2009). Modulation of the action control system by social intention: unexpected social requests override preplanned action. *J. Exp. Psychol. Hum. Percept. Perform.* 35 1490–1500. 10.1037/a0015777 19803652

[B33] Sebanz N. Bekkering H. Knoblich G. (2006). Joint action: bodies and minds moving together. *Trends Cogn. Sci.* 10 70–76. 10.1016/j.tics.2005.12.009 16406326

[B34] Sebanz N. Knoblich G. Prinz W. (2003). Representing others’ actions: just like one’s own? *Cognition* 88 B11–B21. 10.1016/S0010-0277(03)00043-X 12804818

[B35] Vilar L. Araújo D. Davids K. Travassos B. J. (2012). Constraints on competitive performance of attacker-defender dyads in team sports. *Sports Sci.* 30 459–469. 10.1080/02640414.2011.627942 22260194

