# Supplementary material for: Factors affecting athletes’ motor behavior after the observation of scenes of cooperation and competition in competitive sport: the effect of sport attitude
Source: Front Psychol. 2015 Oct 28;6:1648. doi: 10.3389/fpsyg.2015.01648 (PMC4623160; doi:10.3389/fpsyg.2015.01648)
Supplement: Supplementary file 1 [file Table_1.DOC]

Supplementary Table 1

Mixed-design ANOVAs performed on kinematic parameters

The within-subjects factor was type of scene (cooperation vs competition) and the between-subjects factor was participants’ gender (male vs female).

| **Reach time (ms)** | F(1,18)=0.004; p=0.95 |
| --- | --- |
| **Time to peak velocity of reach (ms)** | F(1,18)=0.009; p=0.92 |
| **Peak elevation (mm)** | F(1,18)=0.025; p=0.88 |
| **Grasp time (ms)** | F(1,18)=0.138; p=0.71 |
| **Time to maximal finger aperture (ms)** | F(1,18)=1.064; p=0.32 |
| **Peak velocity of finger opening (mm/s)** | F(1,18)=2.122; p=0.16 |
| **Time to peak velocity of finger opening (ms)** | F(1,18)=0.255; p=0.62 |
| **Maximal Finger Aperture (mm)** | F(1,18)=0.047; p=0.83 |

Mixed-design ANOVAs performed on kinematic parameters

The within-subjects factor was type of scene (cooperation vs competition) and the between-subjects factor was type of sport (basketball vs soccer vs water polo vs volleyball vs rugby).

| **Reach time (ms)** | F(4,15)=0.784; p=0.55 |
| --- | --- |
| **Time to peak velocity of reach (ms)** | F(4,15)=0.133; p=0.97 |
| **Peak elevation (mm)** | F(4,15)=0.417; p=0.79 |
| **Grasp time (ms)** | F(4,15)=0.979; p=0.45 |
| **Time to maximal finger aperture (ms)** | F(1,18)=0.758; p=0.57 |
| **Peak velocity of finger opening (mm/s)** | F(4,15)=1.092; p=0.39 |
| **Time to peak velocity of finger opening (ms)** | F(4,15)=0.583; p=0.68 |
| **Maximal Finger Aperture (mm)** | F(4,15)=1.209; p=0.35 |
