# Supplementary material for: Factors affecting athletes’ motor behavior after the observation of scenes of cooperation and competition in competitive sport: the effect of sport attitude
Source: Front Psychol. 2015 Oct 28;6:1648. doi: 10.3389/fpsyg.2015.01648 (PMC4623160; doi:10.3389/fpsyg.2015.01648)
Supplement: Supplementary file 2 [file Data_Sheet_1.DOC]

Data Sheet 1: Self-evaluation questionnaire

| Age: |
| --- |
| Faculty: |
| **Are you amateur or professional athlete? (How long do you practice team sports)** |
| □ professional (expert): more than 1 years |
| □ amateur (non-expert): less than 1 years |
| **How often do you practice team sports?** |
| □ more than four days a week |
| □ more than three days a week |
| □ more than two days a week |
| □ less than once a week |
| **Which sport do you practice?** |
| □ soccer |
| □ basketball |
| □ water polo |
| □ volleyball |
| □ rugby |
| □ Others (Pls. specify) |
| **What is your role within your team?** |
| □ defender |
| □ attacker |
| □ Others (Pls. specify) |
| **During a match, do you exhibit a competitive (defined as: having a strong desire to compete or to succeed) or cooperative (defined as: working or acting together willingly for a common purpose or benefit) behavior?** |
| □ cooperative |
| □ competitive |
| □ Others (Pls. specify) |
